# Neglected tropical diseases: Need for sensitization of medical students

**DOI:** 10.4103/0253-7613.42309

**Published:** 2008-06

**Authors:** C.S. Gautam, Sangeeta Bhanwra

**Affiliations:** Department of Pharmacology, Government Medical College, Sector-32, Chandigarh -160 030, India. E-mail: doc_sangeeta@yahoo.com

The neglected tropical diseases are a set of thirteen disabling conditions that are among the most common chronic infections in the developing countries like India. About 350 million people are already disabled or severely impaired due to them. Over 3 billion children, women and men are at risk worldwide. These neglected tropical diseases lead to long term disability and the resultant poverty. Seven most prevalent and neglected tropical diseases are ascariasis, trichuriasis, hookworm infection, schistosomiasis, lymphatic filariasis, trachoma and onchocerciasis. Together, these diseases are responsible for billions of infections in the developing world and more than six million deaths per year.[1] Population mostly affected from these diseases are the poorest people in the tropical and subtropical areas of the world. Increased awareness of public as well as the political will are needed to reduce the negative impact of neglected tropical diseases on the health and the social and economic wellbeing of affected communities.[2]

These neglected tropical diseases, have been recently, identified as ‘targets of opportunity’ by WHO and US Centers for Disease Control and Prevention, in an effort to improve global health, while creating more vigorous economies and a better quality of life in some of the world's poorest countries. These diseases are targets of opportunity because through the expanded use of mass drug administration as well as targeted treatments, we now have the opportunity to control or even eliminate some of the most important neglected tropical diseases in terms of prevalence and disease burden.[3] Strategies to control these diseases are based on surveillance, early diagnosis and treatment, vector control in most of these infections, mass drug administration and patient education. The use of mass drug administration for the control of neglected tropical diseases or preventive chemotherapy was first started in China. Prolonged and targeted use of diethylcarbamazine resulted in the elimination of lymphatic filariasis and widespread use of praziquantel, in control of schistosomiasis in that country. This has promoted the concept of international partnership to control or eliminate these infections.

Such partnerships are being formed by pharmaceutical companies mainly through donation of drugs for mass administration. Such comprehensive programmes can be executed in India also.

Lymphatic filariasis (LF) is the second most common vector borne parasitic disease after malaria, found in over 80 tropical and subtropical countries. According to WHO, it is the second most common cause of long term disability after mental illness. One third of people infected with LF live in India.[4] So, a global programme to eliminate LF was launched in 2000. This programme largely depends on mass administration of diethylcarbamazine with or without ivermectin or albendazole. Studies show that integration of vector control with the mass drug administration sustains this programme in a better way and decreases the time required to eliminate LF.[5] Though it is a region-based disease but with the advancement in the connectivity through railways or road the specific boundaries cannot be drawn. Hence, teaching of diseases like LF should not be region based but exhaustive knowledge about these diseases should be given an utmost priority.

In India, where a large number of people are poor, uneducated and underprivileged it should be considered a moral obligation of medical fraternity to address the problems of the poor. The problem needs to be addressed during the formative years in MBBS curriculum. The domain does not belong to Community Medicine alone but an equal importance should be given in Pharmacology training also in a more comprehensive manner. Several steps can be taken to sensitize the medical students in this regard as follows:

1. An appropriate stress must be given to the National Health Programmes right at the beginning of the Pharmacology teaching.
2. Students must be educated about the seven neglected tropical diseases and their management in details. They should be apprised of the fact that if these diseases are tackled at an early stage, some major complications can be avoided.
3. The students should know the importance of keeping in mind the cost of the drugs while prescribing for these diseases.
4. These topics must be given as much importance as the diseases of CNS, CVS, GIT and Respiratory System and should be taken up by the senior faculty.
